# No Danger for Medical Interest and Awareness towards Celiac Disease. Reply to Greenaway et al. Why Is There Medical Inertia and Nihilism to Celiac Disease? Comment on “Pivetta et al. In Elderly Anemic Patients without Endoscopic Signs of Bleeding Are Duodenal Biopsies Always Necessary to Rule Out Celiac Disease? *Diagnostics* 2022, *12*, 678”

**DOI:** 10.3390/diagnostics12071511

**Published:** 2022-06-21

**Authors:** Giulia Pivetta, Emanuele Dilaghi, Edith Lahner

**Affiliations:** Department of Medical-Surgical Sciences and Translational Medicine, Sant’Andrea Hospital, University Sapienza, 00189 Rome, Italy; giulia_pivetta@libero.it (G.P.); emanuele.dilaghi@uniroma1.it (E.D.)

We would like to thank Greenaway et al. [1] for their comment on our recent paper (2) and their interesting point of view to which we reply with pleasure.

Firstly, we would like to specify that in our study population, duodenal intraepithelial lymphocytosis (DIL) was assessed in 26 patients (5.5%), and was very uncommon in the elderly (only one DIL patient was over 70 years of age) [2]. We are well-aware that DIL may be a histological feature of celiac disease (CD) and that only about 16% of DIL patients may be diagnosed with CD. According to guidelines [3], in our center, for all patients with DIL as a duodenal finding, serological screening for CD was proposed to rule out an underlying CD; highly suspicious drugs were withdrawn; intestinal parasitosis, autoimmune comorbidities, and HLA-DQ2/8 were assessed; and, when present, H. pylori infection was treated and endoscopy with biopsies was repeated after eradication treatment to assess eventual regression of DIL. This is why we considered these patients as a separate group, and we can reasonably affirm that the standardized approach to DIL in our center, a referral center for CD, can reduce the misdiagnosis of CD to a negligible percentage [4]. 

Secondly, we fully agree with Greenaway et al. [1] that a delay in the diagnosis of CD, particularly in the elderly, can lead to several complications such as CD-related malignancy and low-impact bone fractures, but also malabsorption of important micronutrients such as iron, vitamin D and others. In our center, we routinely follow up hundreds of CD patients, so we are aware of this issue. As a matter of fact, we would like to point out that in our center, routine duodenal biopsies are obtained during upper gastrointestinal endoscopy in patients presenting with iron-deficiency anemia (IDA) without bleeding, but also in the vast majority of those with dyspepsia or other symptoms. Precisely thanks to this approach were we able to collect the data reported in the cited study [2]. This approach is perfectly in agreement with the former edition of British guidelines which recommended that without manifest bleeding or any other evident cause of IDA, all IDA patients should undergo gastroscopy with duodenal biopsies [5]. However, the revised, more recent guidelines again recommend that in IDA patients, duodenal biopsies should be performed, but only in the case of a positive or unperformed celiac serology [6]. Focusing on this issue, our doubt regarded the diagnostic yield of performing routine duodenal biopsies during the endoscopic evaluation for the presence of IDA in a specific group of elderly patients without overt endoscopic signs of bleeding. In elderly anemic patients without manifest endoscopic signs of bleeding, diagnosis of CD should be less presumable in the face of other, more frequent conditions, such as peptic ulcer, colorectal cancer, and atrophic gastritis [7]. 

Indeed, our study [2] clearly showed that in not-bleeding IDA patients, CD or other duodenal histological alterations significantly decreased with increasing age and were very uncommon in patients over 70 years of age. 

Thirdly, regarding the cost-effectiveness of duodenal biopsies in IDA, an important factor to take into account, the study [8] cited by Greenaway et al. [1] has been already discussed in our paper [2]. In this work [8], the authors estimated the cost-effectiveness considering a group of patients that were younger of age than in our study: a maximum of 60 years in Broide et al. [8] and >70 to 93 years in Pivetta et al. [2].

Finally, as a center with a multiannual experience and expertise in autoimmune and malabsorption diseases, obviously, we are aware of the importance of CD diagnosis, both for the young and the elderly population, without excluding anyone. In our center, we carefully evaluate all patients in toto considering together clinical, serological, endoscopic, and histological features to diagnose or rule out diseases. So, the standardized behavior including duodenal biopsies usually applied in our GI endoscopy unit perfectly keeps in line with your opinion, but what we wanted to assess in our study was whether the routine approach including duodenal biopsies indistinctly to all not-bleeding IDA patients can be considered justified and useful. Our data showed that this approach in the specific subset of very elderly IDA patients without GI bleeding signs could be questionable and be reconsidered due to the low diagnostic yield of duodenal pathology, especially in the absence of macroscopic duodenal alterations or other suspicious signs of related intestinal diseases, while CD can be ruled out by serology when clinically appropriate [2].

In conclusion, we think that the results of our study certainly do not promote medical inertia nor nihilism to CD. On the contrary, we are confident that every effort of active research on CD increases medical interest and consciousness of this widespread condition. Further, we are also convinced that even conflicting opinions and contrasting points of view on a specific research issue do not automatically mean danger for medical awareness, but, on the contrary, may contribute to ultimately increasing knowledge and better care for patients.

## References

[1] Greenaway E.A., Raju S.A., Sanders D.S. (2022). Why Is There Medical Inertia and Nihilism to Celiac Disease? Comment on Pivetta et al. In Elderly Anemic Patients without Endoscopic Signs of Bleeding Are Duodenal Biopsies Always Necessary to Rule out Celiac Disease? *Diagnostics* 2022, *12*, 678. Diagnostics.

[2] Pivetta G., Coluccio C., Dilaghi E., Lahner E., Pilozzi E., Carabotti M., Corleto V.D. (2022). In Elderly Anemic Patients without Endoscopic Signs of Bleeding Are Duodenal Biopsies Always Necessary to Rule Out Celiac Disease?. Diagnostics.

[3] Al-Toma A., Volta U., Auricchio R., Castillejo G., Sanders D.S., Cellier C., Mulder C.J., Lundin K.E.A. (2019). European Society for the Study of Coeliac Disease (ESsCD) guideline for coeliac disease and other gluten-related disorders. United Eur. Gastroenterol. J..

[4] Galli G., Purchiaroni F., Lahner E., Sacchi M.C., Pilozzi E., Corleto V.D., Di Giulio E., Annibale B. (2017). Time trend occurrence of duodenal intraepithelial lymphocytosis and celiac disease in an open access endoscopic population. United Eur. Gastroenterol. J..

[5] Goddard A.F., McIntyre A.S., Scott B.B. (2000). Guidelines for the management of iron deficiency anaemia. Gut.

[6] Goddard A.F., James M.W., McIntyre A.S., Scott B.B. (2011). Guidelines for the management of iron deficiency anaemia. Gut.

[7] Annibale B., Capurso G., Chistolini A., D’Ambra G., DiGiulio E., Monarca B., DelleFave G. (2001). Gastrointestinal causes of refractory iron deficiency anemia in patients without gastrointestinal symptoms. Am. J. Med..

[8] Broide E., Matalon S., Kriger-Sharabi O., Richter V., Shirin H., Leshno M. (2016). Cost effectiveness of routine duodenal biopsies in iron deficiency anemia. World J. Gastroenterol..

